# Combined Effect of Fatty Diet and Cognitive Decline on Brain Metabolism, Food Intake, Body Weight, and Counteraction by Intranasal Insulin Therapy in 3×Tg Mice

**DOI:** 10.3389/fncel.2019.00188

**Published:** 2019-05-03

**Authors:** Elena Sanguinetti, Maria Angela Guzzardi, Daniele Panetta, Maria Tripodi, Vincenzo De Sena, Mauro Quaglierini, Silvia Burchielli, Piero A. Salvadori, Patricia Iozzo

**Affiliations:** ^1^Institute of Clinical Physiology, National Research Council (CNR), Pisa, Italy; ^2^Scuola Superiore di Studi Universitari Sant’Anna, Pisa, Italy; ^3^Fondazione Toscana Gabriele Monasterio (FTGM), Pisa, Italy

**Keywords:** positron emission tomography, high-fat diet, Alzheimer’s disease, triple transgenic mice, cognitive disease, cerebral glucose uptake and insulin resistance, PAI-1, adipokines

## Abstract

Obesity and cognitive decline can occur in association. Brain dysmetabolism and insulin resistance might be common underlying traits. We aimed to examine the effect of high-fat diet (HFD) on cognitive decline, and of cognitive impairment on food intake and body-weight, and explore efficacy of chronic intranasal insulin (INI) therapy. We used control (C) and triple transgenic mice (3×Tg, a model of Alzheimer’s pathology) to measure cerebral mass, glucose metabolism, and the metabolic response to acute INI administration (cerebral insulin sensitivity). Y-Maze, positron emission-computed tomography, and histology were employed in 8 and 14-month-old mice, receiving normal diet (ND) or HFD. Chronic INI therapy was tested in an additional 3×Tg-HFD group. The 3×Tg groups overate, and had lower body-weight, but similar BMI, than diet-matched controls. Cognitive decline was progressive from HFD to 3×Tg-ND to 3×Tg-HFD. At 8 months, brain fasting glucose uptake (GU) was increased by C-HFD, and this effect was blunted in 3×Tg-HFD mice, also showing brain insulin resistance. Brain mass was reduced in 3×Tg mice at 14 months. Dentate gyrus dimensions paralleled cognitive findings. Chronic INI preserved cognition, dentate gyrus and metabolism, reducing food intake, and body weight in 3×Tg-HFD mice. Peripherally, leptin was suppressed and PAI-1 elevated in 3×Tg mice, correlating inversely with cerebral GU. In conclusion, 3×Tg background and HFD exert additive (genes*lifestyle) detriment to the brain, and cognitive dysfunction is accompanied by increased food intake in 3×Tg mice. PAI-1 levels and leptin deficiency were identified as potential peripheral contributors. Chronic INI improved peripheral and central outcomes.

## Introduction

Obesity and neurodegenerative diseases are growing in prevalence. In humans, obesity was shown to predict cognitive impairment (Elias et al., 2005; Whitmer et al., 2005; Hassing et al., 2010), associating with cerebral matter losses (Driscoll et al., 2012), which were reversed by dieting (Haltia et al., 2007). In obese rodents, these abnormalities were related to, e.g., reduced hippocampal plasticity (Wu et al., 2003). In APP/PS1 double transgenic mice (with features of Alzheimer’s disease), diet-induced obesity worsened amyloid burden and cognitive performance (Cao et al., 2007). In turn, there is also evidence that the damage to cognitive areas can lead to overeating (Davidson et al., 2009), and that the central nervous system exerts control on peripheral glucose homeostasis (Obici et al., 2002; Heni et al., 2017).

Among common underlying factors, cerebral insulin resistance has gained attention. Insulin promotes neuronal growth and differentiation, and amyloid and tau processing (Steen et al., 2005), protecting learning and memory (Zhao and Alkon, 2001), and defects in cerebral insulin receptors or action have been observed in patients with Alzheimer’s disease (Zhao and Alkon, 2001; Talbot et al., 2012). In high-fat and/or high-fructose fed animals, defects in insulin action are accompanied by alterations in synaptic, dendritic, hippocampal integrity, and cognitive dysfunction (Winocur and Greenwood, 2005; Stranahan et al., 2008; Arnold et al., 2014; Calvo-Ochoa et al., 2014). On the other side, brain insulin signaling plays a critical role in the maintenance of energy balance, food intake, and weight gain (Stockhorst et al., 2004; Stranahan et al., 2008; Fronczek et al., 2012; Jauch-Chara et al., 2012; Madden et al., 2012; Talbot et al., 2012). INI treatment has shown promise as therapy to overcome cerebral insulin resistance in dementia and in obesity (Benedict et al., 2004; Craft et al., 2012; Jauch-Chara et al., 2012; Ott et al., 2012; Heni et al., 2017; Nedelcovych et al., 2018). However, there is much heterogeneity in the animal models and intervention protocols used, and in the level of efficacy attained. The current evidence suggests that short-term intra-cerebral insulin therapy results in cognitive improvements (Park et al., 2000; Marks et al., 2009; Vandal et al., 2014; Salameh et al., 2015), but efficacy of protracted insulin regimens has been less explored and not confirmed in most of the existing studies, in which phenomena of desensitization due to overdosing have been advocated (Kamal et al., 2012; Nazarians-Armavil et al., 2013; Bell and Fadool, 2017).

In this study, we tested the hypotheses that (a) high-fat feeding affects cognition, brain mass, cerebral metabolism, and cerebral insulin sensitivity, especially in genetically predisposed subjects, (b) regulation of food intake and body weight is compromised in subjects with impaired cognition, and (c) chronic INI prevents these effects. We used C and a 3×Tg mouse model of Alzheimer’s type pathology fed high fat (HFD) or ND. An exploratory evaluation of efficacy of chronic INI to reverse the observed phenotype was carried out in the group showing more severe conditions, i.e., 3×Tg-HFD mice.

## Materials and Methods

### Study Design

**FIGURE 1:**
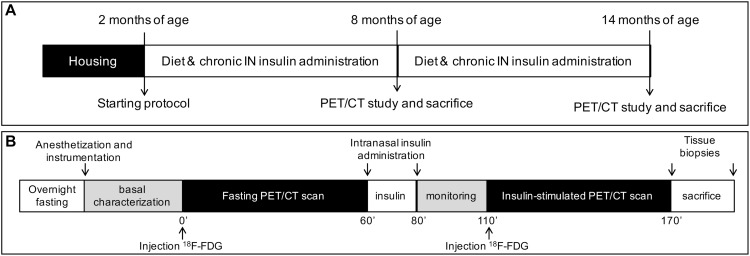
The panels show the study design **(A)**, and PET-CT scanning session **(B)**. ND and HFD were tested in controls and 3×Tg mice, whereas chronic intranasal insulin therapy (INI) was tested in an additional group of 3×Tg-HFD mice.

We studied 145 male mice, including *n* = 76 controls (B6129SF2/J, strain# 101045) and *n* = 69 3×Tg mice (B6;129-Psen1^tm1Mpm^Tg(APPSwe,tauP301L)1Lfa/Mmjax; strain# 004807, The Jackson Laboratory, Bar Harbor, ME, United States). In a subset of 8 months old animals, microbiome-metabolome signatures associated with 3×Tg background, brain glucose extraction and HFD were recently published (Sanguinetti et al., 2018). The study design is summarized in Figures 1A,B. Animals were housed under 12-h light/12-h dark cycles and controlled room temperature (22°C), with *ad libitum* access to food and fresh water. Mice were divided in five groups: (1) ND (C-ND, B6129SF2J, *n* = 37, 11% kcals from fat, Mucedola, Milan, Italy); (2) high-fat diet (C-HFD, B6129SF2J, *n* = 39, 58% kcals from fat); (3) 3×Tg-ND (*n* = 22); (4) 3×Tg-HFD (*n* = 22); (5) 3×Tg-HFD and chronic INI (3×Tg-HFD + INI, *n* = 25). Diets and INI were started at 2 months of age. Body weight and food intake were monitored weekly, and random glycaemia every ∼7 weeks. At 8 and 14 ± 1 months of age, cognitive performance was measured by Y-maze test (Panlab, Harvard Apparatus, Barcelona, Spain), and positron emission and CT with ^18^FDG (IRIS PET/CT, Inviscan SAS, Strasbourg, France) was performed in a subset of 85 mice. At the end of *in vivo* procedures, animals were euthanized, and the brain collected and weighted. The experimental protocol was conducted under the D.L.116/92 implementation of European Economic Community directive 609/86 regarding the protection of animals used for experimental and other scientific purposes.

### Chronic Insulin Therapy

This explorative study was carried out in 3×Tg-HFD-INI mice. Weak sedation by 1–2% (v/v) isoflurane (IsoFlo^®^, Abbott Laboratories, Chicago, IL, United States) was used until correct positioning, and INI delivery was carried out in awake mice, under neck extension (Marks et al., 2009; Sanguinetti et al., 2018). INI was administered daily for 1 week, and weekly thereafter, to minimize desensitization or adverse effects (Kamal et al., 2012; Nazarians-Armavil et al., 2013; Anderson et al., 2017), accounting for the notion that each insulin dose has persisting effects over days (Meredith et al., 2015; Salameh et al., 2015). Each INI administration consisted of 0.87 UI in 24 μl vehicle solution (PBS, Sigma-Aldrich, St Louis, MO, United States), as delivered by pipette in four 6-μl drops, alternating nares every 1 min to ensure fluid inhalation (Marks et al., 2009).

### Y-Maze Test

Cognitive performance and explorative behavior were measured by spontaneous alternation testing in a standard 3-arm Y-maze (Panlab, Harvard Apparatus, Barcelona, Spain) during an 8 min session. The test was performed at least 48 h after insulin administration in order to capture the effect of the chronic insulin therapy and to avoid any acute insulin effect. A visual automatic tracking system (Panlab, Harvard Apparatus, Barcelona, Spain) was used to measure: latency time (until first arm choice), spontaneous alternation triplets (number of three consecutive entries in different arms), percentage of alternation triplets (against the maximum possible number), zone transition number, and total arm entries. Resting time, traveled distance, and speed were also measured.

### PET-CT Scanning

The imaging session is shown in Figure 1B. Anesthesia was induced in fasted mice by 3–4% (v/v), and maintained with 1–2% (v/v) isoflurane. A rectal probe was positioned, and baseline temperature measured. Then, a heated pad was used to prevent the decline in body temperature due to anesthesia. Mice were positioned in a μPET-CT tomograph (IRIS PET/CT, Inviscan SAS, Strasbourg, France) and CT scans were acquired. Then, two 60-min PET scans were performed after i.p. ^18^FDG injection (7.6 ± 0.1 and 7.9 ± 0.1 MBq, first and second scan), one in the fasted state, and one after 30-min of an acute INI dose (0.87 UI in 24 μl) (Marks et al., 2009) (Figure 1B). Glycemia was monitored in tail blood.

### PET-CT Image Processing

Positron emission tomography data were corrected for dead time and radioactive decay, reconstructed by standard algorithms, and co-registered to CT images by AMIDE Medical Image Data Examiner 1.0.5. Volumes of interest were drawn in brain images corresponding to frontal, somatosensory and temporal cortices, dorsal striatum, globus pallidus, thalamus, hypothalamus, amygdala, hippocampus. Fractional tracer extraction (FE), reflecting the intrinsic ability of the brain to actively extract glucose from the circulation (Thie, 1995), was expressed as ratio of tissue activity to the ID per gram of body weight (% ID/g). GU, resulting from the combined effects of FE and glucose delivery from blood, was computed as product of % ID/g and glycaemia during imaging (Thie, 1995).

### Circulating Markers

After the imaging session, animals were euthanized and blood was collected and centrifuged 10 min at 4000 rpm. Plasma concentrations of insulin, leptin, interleukin-6 (IL-6), tumor necrosis factor-α (TNF-α), resistin, plasminogen activator inhibitor-1 (PAI-1), and monocyte chemoattractant protein-1 (MCP-1) were measured by Luminex^®^ xMAP^®^ technology (Mouse Adipokine Magnetic Bead Panel, Merck-Millipore Corp., MO, United States), according to manufacturer’s instructions.

### Brain Histology

Surface of the dentate gyrus, and thickness of the granule cell layer were measured in histological sections. After sacrifice, the brain was dissected, and one hemisphere was fixed in 10% neutral-buffered formalin (20–24 h), and processed for paraffin-embedding. Sections were cut on a coronal plane at a thickness of 8 μm on a rotary microtome and mounted on clean glass Polysine^TM^ slides (Menzel-Gläser, Germany). Sections were stained with hematoxylin-eosin (Bio-Optica, Italy), according to standard protocol. Each section was documented at 5, 10, and 40× magnification using a Axioskop optical microscope connected with an AxioCam MRc5 color-camera and AxioVision analysis software (Carl Zeiss, Germany). By using the ImageJ software (version 2.0.0-rc-43/1.51k^1^), contour of the dentate gyrus was manually drawn in 10× images and its area recorded. Thickness of the granule cell layer of the suprapyramidal and of the infrapyramidal blades was based on the average of three measures obtained in proximity of the apex, the mid and the distal parts in each blade.

### Statistical Analysis

Data are presented as mean ± SEM. Statistical analyses were performed by IBM^®^ SPSS^®^ Statistics for Mac OS X (version 24.0, Chicago, IL, United States). Data distribution was assessed by Shapiro-Wilk test. For normally distributed variables and variables that were normalized by logarithmic transformation (e.g., anthropometrics, cognitive parameters, and plasma levels of circulating markers), group comparisons were performed by ANOVA, with statistical significance localized by Fisher’s least significant difference (LSD) *post hoc* analysis, or by two-tailed T-test. Mann-Whitney U test was used when normal distribution was not achieved by logarithmic transformation (i.e., PET data). Analysis of intra-group changes in brain GU from fasting to acute insulin delivery was performed by Wilcoxon test. Two-way ANOVA was also used to confirm independent effects of diet and genotype, and their interaction, and brain metabolic responses to acute insulin administration (mixed design ANOVA). Statistical significance was set at *p*-value ≤ 0.05.

## Results

Table 1 provides sample sizes per group and time-point, Tables 2, 3 and Supplementary Tables S1, S2 summarize two-way ANOVA and mixed-design ANOVA analyses.

**TABLE 1:** Sample size referring to results shown in figures and tables.

	C-ND	C-HFD	3×Tg-ND	3×Tg-HFD	3×Tg-HFD + INI
** 8 months**
Body weight (20–24w)(g)	37	39	22	22	25
Random glycemia (21-22w) (mmol/l)	35	39	21	20	24
Caloric Intake (20-24w) (Kcal)	37	39	22	22	25
Circulating cytokines	9–11	10–12	8	6–7	10–11
Cognitive variables	39	39	21	20	23
Brain mass	5	5	4	4	7
Brain histology	3	5	3	3	5
Brain PET baseline	7	9	8	7	11
Brain PET + acute insulin	7	9	8	7	10
**14 months**
Body weight (44w) (g)	14	16	11	11	8
Random glycemia (42–44w) (mmol/l)	14	16	11	11	8
Caloric Intake (Kcal)	14	16	11	11	8
Circulating cytokines	11–13	14–16	8–9	5–6	7
Cognitive variables	16	14	9	7	7
Brain mass	6	6	9	6	7
Brain histology	6	6	4	4	4
Brain PET baseline	10	12	8	6	7
Brain PET + acute insulin	10	11	8	5	7

**TABLE 2:** Two-way ANOVA for the effects of diet and genotype in group comparisons.

	Diet	Genotype	Diet* Genotype
**8 months**
Body weight (g)	**<0.0005**	**<0.0005**	0.793
Body mass index (kg/m^2^)	**<0.0005**	0.108	0.990
AUC body weight (g*time)	**0.015**	**<0.0005**	0.961
Random glycemia (mmol/l)(21-22w)	**0.001**	**<0.0005**	**0.035**
Insulin (pg/ml)	0.952	0.806	0.153
Leptin (pg/ml)	**0.039**	**<0.0005**	**0.036**
Resistin (pg/ml)	**0.045**	0.906	0.297
IL-6 (pg/ml)	0.937	0.187	0.724
TNF-alpha (pg/ml)	0.343	0.791	0.672
MCP-1 (pg/ml)	0.580	**0.004**	0.561
PAI-1 (pg/ml)	0.204	**0.001**	0.555
Alternation triplets (n)	0.125	**<0.005**	0.659
Alternation triplets (%)	0.143	**<0.005**	0.401
Resting time (s)	0.416	0.248	0.437
Total arm entries (n)	0.444	0.353	0.052
Total distance (cm)	0.133	0.106	0.259
Speed (cm/s)	0.141	0.106	0.252
Latency time (s)	0.825	0.293	0.946
Zone transition (n)	0.340	0.252	0.080
Brain mass (g)	0.333	0.239	0.710
Cerebral SUV (% ID/g) baseline	0.528	**<0.005**	0.849
Cerebral SUV (% ID/g) + insulin	0.434	**<0.005**	0.756
Cerebral GU (% ID/g * mmol/l) baseline	**<0.005**	0.067	0.351
Cerebral GU (% ID/g * mmol/l) + insulin	**<0.005**	0.593	0.477
**14 months**			
Body weight (g)	**0.004**	**0.001**	0.214
Body mass index (kg/m^2^)	**0.011**	0.658	0.167
AUC body weight (g*time)	**0.011**	**<0.0005**	0.230
Random glycemia (mmol/l)	**0.003**	**<0.0005**	0.796
Insulin (pg/ml)	0.182	**0.004**	0.092
Leptin (pg/ml)	**0.026**	**<0.0005**	0.178
Resistin (pg/ml)	**0.022**	**0.025**	0.131
IL-6 (pg/ml)	0.543	**<0.0005**	0.063
TNF-alpha (pg/ml)	0.060	0.384	0.162
MCP-1 (pg/ml)	0.669	**0.005**	0.576
PAI-1 (pg/ml)	0.589	**0.029**	0.460
Alternation triplet (n)	0.120	**<0.0005**	0.495
Alternation triplet (%)	0.727	**0.001**	0.796
Resting time (s)	0.127	**<0.005**	0.154
Total arm entries (n)	**0.004**	0.085	0.079
Total distance (cm)	0.091	**<0.0005**	0.222
Speed (cm/s)	0.100	**<0.0005**	0.241
Latency time (s)	0.188	0.054	0.300
Zone transition (n)	**0.004**	0.077	0.083
Brain mass (g)	0.732	**<0.005**	0.270
Cerebral SUV (% ID/g) baseline	0.346	**0.001**	0.871
Cerebral SUV (% ID/g) + insulin	0.824	**0.001**	0.318
Cerebral GU (% ID/g * mmol/l) baseline	0.511	0.136	0.827
Cerebral GU (% ID/g * mmol/l) + insulin	0.268	0.607	0.644

*In bold are indicated significant *p*-values.*

**TABLE 3:** Mixed design ANOVA for the effects of acute intranasal insulin stimulus.

	Acute insulin	Acute insulin * diet	Acute insulin * genotype	Acute insulin * diet * genotype
**Cerebral fractional extraction**				
8 months	0.359	0.927	0.794	0.878
14 months	0.329	**0.024**	0.814	0.102
**Cerebral glucose uptake**				
8 months	**<0.0005**	0.740	**0.001**	**0.011**
14 months	**<0.0005**	0.755	0.222	0.107

*In bold are indicated significant *p*-values.*

**FIGURE 2:**
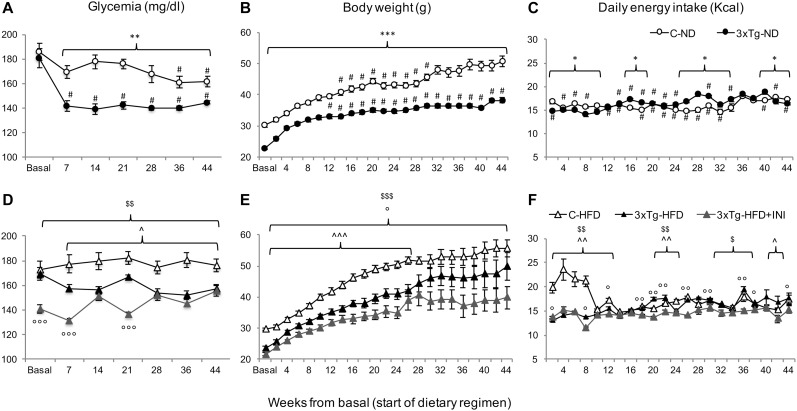
The figure documents lower glycemia **(A,D)** and body weight **(B,E)**, and higher energy intake **(C,F)** in 3×Tg compared to diet-matched controls, with an effect of chronic INI to reduce all parameters **(D–F)**. Data are presented as mean ± SEM. Sample sizes are given in Table 1. Panels **A–F** *p < 0.05, **p < 0.01, ***p < 0.001 between groups (within-panel), ^#^*p* < 0.05 ND **(A–C)** vs. HFD **(D–F)** within-strain (between panels), panels **D–F** ${}^{\wedge }$*p* < 0.05 or less (${}^{\wedge }$${}^{\wedge }$) C-HFD vs. 3×Tg-HFD, ^$^*p* < 0.01 C-HFD vs. 3×Tg-HFD + INI, °*p* < 0.05 or less (°°,°°°) 3×Tg-HFD vs. 3×Tg-HFD + INI.

**FIGURE 3:**
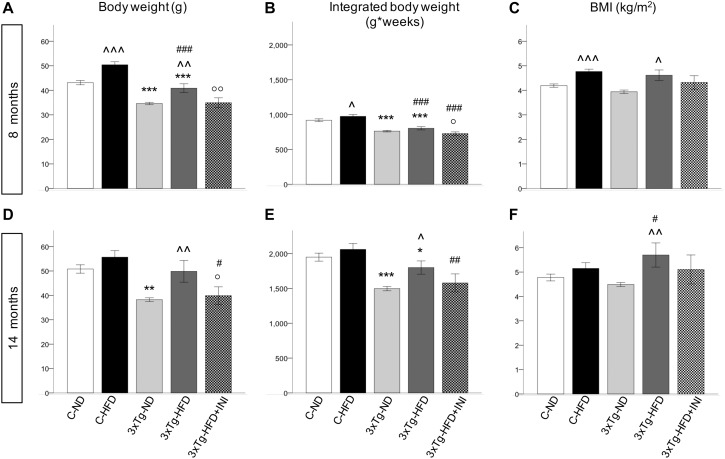
Body weight, areas under the curves and BMI achieved by the study groups at final time-points of 8 **(A–C)** and 14 months of age **(D–F)**, showing that body weight was affected by diet (positively) and by 3×Tg background (negatively), whereas BMI was only dependent on diet, since 3×Tg mice were lighter, but also shorter (data not shown) than controls. Data are presented as mean ± SEM. Sample sizes are given in Table 1. *p < 0.05 or less (**,***) ND-ND and HFD-HFD (between strains), ${}^{\wedge }$*p* < 0.05 or less (${}^{\wedge }$${}^{\wedge }$,${}^{\wedge }$${}^{\wedge }$${}^{\wedge }$) HFD vs. ND (within-strain), ^#^*p* < 0.05 or less (^#⁢#^,^#⁢#⁢#^) vs. ND control group (reference group), °*p* < 0.05 or less (°°) for treated 3×Tg-HFD + INI vs. untreated 3×Tg-HFD.

**FIGURE 4:**
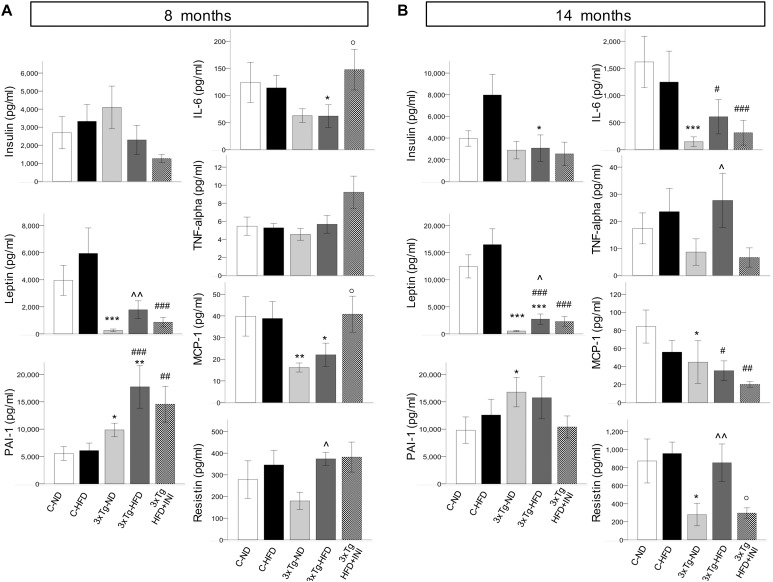
Plasma levels of cytokines and adipokines measured in each study group at final time-points of 8 **(A)** and 14 months of age **(B)**. Data are presented as mean ± SEM (non-normally distributed data were normalized with logarithmic transformation, and parametric tests were used). Sample sizes are given in Table 1. *p < 0.05 or less (**,***) ND-ND and HFD-HFD (between strains), ${}^{\wedge }$*p* < 0.05 or less (${}^{\wedge }$${}^{\wedge }$) HFD vs. ND (within-strain), ^#^*p* < 0.05 or less (^#⁢#^,^#⁢#⁢#^) vs. ND control group (reference group), °*p* < 0.05 for treated 3×Tg-HFD + INI vs. untreated 3×Tg-HFD.

**FIGURE 5:**
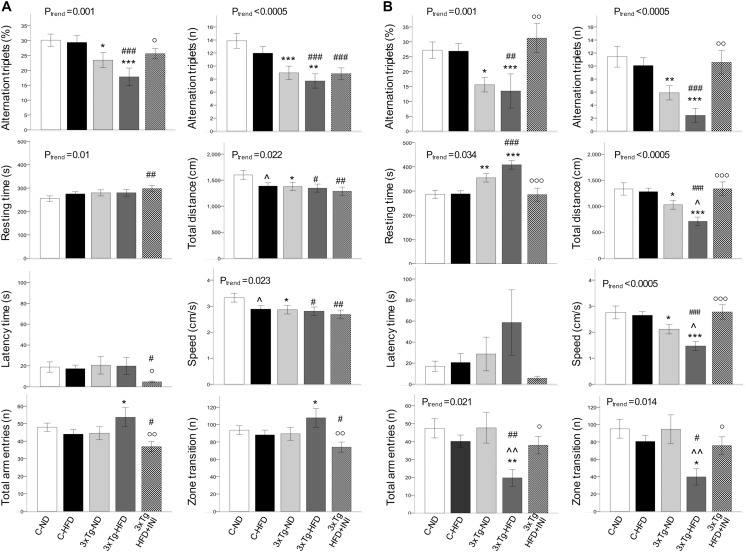
Cognitive function parameters at the age of 8 **(A)** and 14 months **(B)**. Data are presented as mean ± SEM. Sample sizes are given in Table 1. P_trend_ refers to regression analyses across the first four groups. *p < 0.05 or less (**,***) ND-ND and HFD-HFD (between strains), ${}^{\wedge }$*p* < 0.05 or less (${}^{\wedge }$${}^{\wedge }$) HFD vs. ND (within-strain), ^#^*p* < 0.05 or less (^#⁢#^,^#⁢#⁢#^) vs. ND control group (reference group), °*p* < 0.05 or less (°°,°°°) for treated 3×Tg-HFD + INI vs. untreated 3×Tg-HFD.

C-HFD and 3×Tg-HFD mice consumed greater amounts of calories, gained more weight, and had greater glucose levels compared to C-ND and 3×Tg-ND mice. However, compared to diet-matched C, 3×Tg (either -ND or -HFD) mice ate larger amounts of calories, despite lower body weight, normal BMI and lower random glycaemia (Figures 2A–F, 3A–F). Leptin levels were strikingly suppressed in 3×Tg models, with a small counteracting effect of HFD. PAI-1 levels were especially high in 3×Tg-HFD at 8 months (Figure 4A), but also elevated in 3×Tg-ND. MCP1 and IL6 were reduced in 3×Tg models at 8 and 14 months, and TNF-α and resistin levels also tended to be deficient in 3×Tg-ND (Figures 4A,B). In 3×Tg-HFD + INI, energy intake, body weight, and glycaemia were lower than in untreated 3×Tg-HFD mice, though the effect on glycemia was transient (Figures 2D–F). INI also modified inflammatory indices toward normal levels at 8 months, and progressively normalized PAI-1 levels (Figures 4A,B), paralleling the degree of preservation of cognitive function (Figures 5A,B).

### Cognitive Function

In 8 months old mice (Figure 5A) a significant trend toward cognitive dysfunction was found across groups, from C-ND to C-HFD to 3×Tg-ND to 3×Tg-HFD. Spontaneous alternation triplets, both as total count and percentage, were significantly reduced in 3×Tg-ND and 3×Tg-HFD than control groups. These features were more pronounced at 14 months of age (Figure 5B), also involving total arm entries and zone transition number in 3×Tg-HFD (Figure 5B). Significant trends across the four groups, and group differences were seen in resting time, total distance, and speed at both 8 and 14 months (Figures 5A,B). In 14 months groups, reductions in traveled distance and speed and the increase in resting time were significant in 3×Tg-ND compared to ND controls, and severe in 3×Tg-HFD mice vs. all groups (Figure 5B). Interaction analyses (Table 2) confirmed a significant independent effect of genetic model (and a tendency of diet) on cognitive function.

In animals treated with chronic INI, cognitive performance was similar compared to control groups, and significantly higher than in age-matched untreated 3×Tg-HFD mice at 8 months (Figure 5A), and even more (3-folds) at 14 months of age (Figure 5B).

### Brain Glucose Metabolism

**FIGURE 6:**
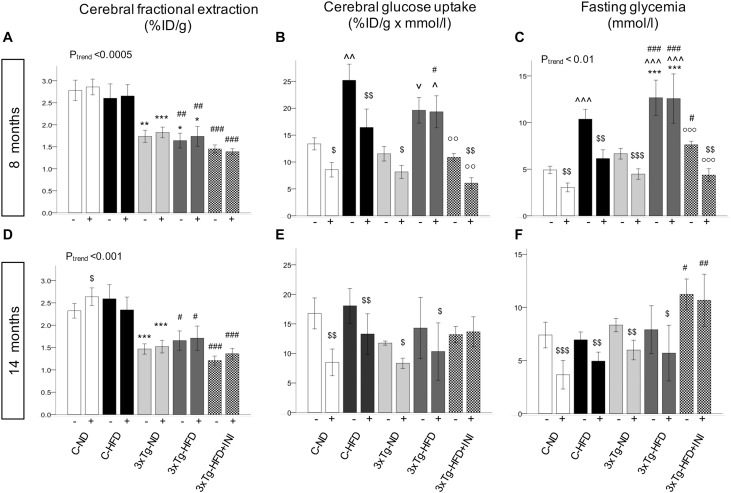
Brain glucose metabolism in 8 (top) and 14 (bottom) months old mice. Fractional extraction **(A,D)** reflects the intrinsic ability of the brain to actively extract glucose from the circulation, and glucose uptake **(B,E)** takes into account the mass effect of circulating glucose, and was computed as product of fractional extraction **(A,D)** and glycaemia during imaging **(C,F)**. Data are presented as mean ± SEM. Sample sizes are given in Table 1. P_trend_ refers to regression analyses across the first four groups. *p < 0.05 or less (**,***) ND-ND and HFD-HFD (between strains), ^∧^*p* < 0.05 or ^v^*p* = 0.064 HFD vs. ND (within-strain), ^#^*p* < 0.05 or less (^#⁢#^,^#⁢#⁢#^) vs. ND control group (reference group), ${}^{\circ \circ }$*p* < 0.01 or less (°°°) for treated 3×Tg-HFD + INI vs. untreated 3×Tg-HFD, ^$^*p* < 0.05 acute intranasal insulin vs. baseline PET scans (within-group, paired tests).

Brain glucose metabolism showed a diffuse reduction in the whole brain (Figures 6A,D) and in all cerebral regions in fractional extraction of [^18^F]FDG (Supplementary Figures S1, S2) in 3×Tg-ND and 3×Tg-HFD mice compared to age-matched controls. An independent effect of 3×Tg genotype to decrease glucose fractional extraction was confirmed in interaction analyses (Table 2). HFD consumption per se did not aggravate, and INI did not alleviate this defect. Cerebral GU (extraction*glycemia) (Figures 6B,C,E,F and Supplementary Figures S3, S4) was greater in all brain regions in HFD compared to ND groups at 8 months, and this effect was blunted by 3×Tg background. Interaction analyses confirmed the independent effect of diet on cerebral GU, and a contrasting effect of 3×Tg background in selected brain regions (Table 2 and Supplementary Table S1). At 8 months, acute INI lowered brain GU, suppressing peripheral glycemia in C-ND, C-HFD and 3×Tg-ND mice (Figures 6B,C, Table 3, and Supplementary Table S2), whereas no effect was observed in 3×Tg-HFD mice (indicative of brain insulin resistance in this group). Chronic INI in 3×Tg-HFD + INI mice was able to reduce the excess in brain GU seen in 3×Tg-HFD mice, and re-establish a normal acute response to insulin at 8 months of age (Figures 6B,C), although cerebral insulin resistance was present at 14 months (Figures 6E,F), consistent with raising blood glucose levels (Figures 1D, 6F).

Among peripheral factors, PAI-1 levels were inversely related to brain GU in pooled age groups, and in 14 months old mice, during fasting (*r* = −0.20, *p* = 0.058, *r* = −0.28, *p* = 0.033) and acute insulin administration (*r* = −0.21, *p* = 0.047, *r* = −0.22, *p* = 0.014).

### Brain Mass and Histology

**FIGURE 7:**
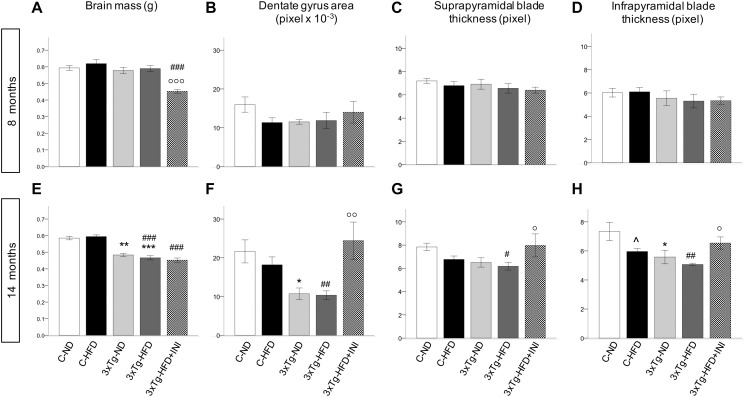
Cerebral morphologicalcharacterization in 8 (top) and 14 (bottom) months old mice, including brain mass **(A,E)**, area of dentate gyrus **(B,F)**, and thickness of suprapyramidal **(C,G)** and infrapyramidal blades **(D,H)**, as measured in coronal brain sections stained with haematoxylin-eosin. Sample sizes are given in Table 1. *p < 0.05 or less (**,***) ND-ND and HFD-HFD (between strains), ${}^{\wedge }$*p* < 0.05 HFD vs. ND (within-strain), ^#^*p* < 0.05 or less (^#⁢#^,^#⁢#⁢#^) vs. ND control group (reference group), °*p* < 0.05 or less (°°, °°°) for treated 3×Tg-HFD + INI vs. untreated 3×Tg-HFD.

Brain mass was significantly reduced in all 3×Tg models at 14 months (Figures 7A–E) and was not modified by chronic INI. The dimension of the dentate gyrus, and the thickness granule cell layer of both suprapyramidal and infrapyramidal blades showed not significant differences in 8 months old HFD or 3×Tg mice (Figures 7B–D). At 14 months (Figures 7F–H), HFD mice had lower infrapyramidal layer thickness, whereas the 3×Tg mice showed a reduction in dentate gyrus area and infrapyramidal layer thickness compared to C-ND mice. In 3×Tg-HFD mice, all measures (dentate gyrus area, suprapyramidal, and infrapyramidal layer thickness) were defective. Chronic INI resulted in normal dimensions of dentate gyrus area, suprapyramidal, and infrapyramidal layer thickness, which were significantly larger in 3×Tg-HFD-INI than in 3×Tg-HFD mice, and comparable to C-ND mice.

In addition, at 14 months dentate gyrus and infrapyramidal layer dimensions were correlated with the percentage of alternation triplets, as assessed by Y maze (*R* = 0.466 and 0.467, *p* = 0.029). The dentate gyrus area was also significantly associated with resting time (*R* = −0.466, *p* = 0.029), total traveled distance (*R* = 0.439, *p* = 0.041), and speed (*R* = 0.447, *p* = 0.037).

## Discussion

This study documents a progressive reduction in cognitive function due to 3×Tg-background, HFD, and their combination. The former had stronger independent influence on alternation triplets, whereas the diet had more impact on the number of entries in Y-Maze arms. As a result, mice in the 3×Tg-HFD group presented worse profiles in all cognitive variables, especially at the age of 14 months. These results are consistent with previous evidence (Knight et al., 2014; Sah et al., 2017) pointing to additive mechanisms, i.e., amyloid-pathology (insoluble Aβ plaque area, tau neuropathology), restricted to 3×Tg mice, and an increase in soluble Aβ40-42 (Vandal et al., 2014), microglia activation (Knight et al., 2014), neuronal oxidative stress and apoptosis (Sah et al., 2017) also induced by HFD. Though it may be argued that the defects in speed and traveled distance seen in our 3×Tg mice can influence the interpretation of cognitive results, the decline in alternation triplets observed with Y-Maze testing was shown to reflect the outcomes of other tests (Morris water maze, smell recognition, novel object recognition tests) in non-Tg and 3×Tg mice fed ND or HFD (Knight et al., 2012; Sah et al., 2017). In fact, the authors of those studies concluded that regardless of the test used, HFD impairs both spatial and non-spatial memory (Knight et al., 2012). To further support our cognitive findings, we measured the surface of the dentate gyrus and thickness of the granule cell layer of the infra- and suprapyramidal blades in histological sections. These are the most affected hippocampal subfields during the development of Alzheimer’s disease and show the greatest amyloid burden in the PDAPP (transgenic for the amyloid precursor protein) mouse model (Reilly et al., 2003). The reduction in dentate gyrus and granule cell layer dimensions did not achieve significance in 8 months old HFD and 3×Tg mice, showing only a tendency. They became significant in 14 months old HFD (infrapyramidal layer thickness) and 3×Tg-ND mice (dentate gyrus area and infrapyramidal layer thickness), and were most severely affected in 3×Tg-HFD mice (dentate gyrus area, suprapyramidal, and infrapyramidal layer thickness), correlating with cognitive findings. These timelines are consistent with the notion that the decline in dentate gyrus neurogenesis becomes measurable at 9 months, and is more evident at 12 months of age in 3×Tg compared to C (Rodriguez et al., 2008).

Considering that glucose is the main cerebral energy substrate, and given the growing implication of cerebral insulin resistance in the pathogenesis of Alzheimer’s disease (Zhao and Alkon, 2001; Steen et al., 2005; Winocur and Greenwood, 2005; Stranahan et al., 2008; Talbot et al., 2012; Arnold et al., 2014; Calvo-Ochoa et al., 2014), we examined for the first time the combined effects of diet and 3×Tg background on three processes underlying brain glucose metabolism *in vivo* (fractional extraction, FE, glucose uptake, GU during fasting, and their response to insulin), as determined by consecutive imaging sessions (fasting, acute INI delivery). First, we observed that the fractional extraction, i.e., the intrinsic capability of the brain to extract FDG from the circulation (Thie, 1995), was 30–40% deficient in our 3×Tg mice, regardless of diet. This defect is typically related to neuronal loss in patients with mild to severe cognitive dysfunction (Silverman et al., 2001; Ossenkoppele et al., 2012), and supports the translational potential of the current mouse model. Second, we found that cerebral GU, representing the actual glucose load entering the tissue (Ashraf et al., 2015) was in excess of 85% in HFD compared to ND groups, but this effect was reduced (especially in temporal cortex, globus pallidus, thalamus, somatosensory cortex) in 3×Tg-HFD mice. In human patients with mild cognitive impairment, cerebral hypermetabolism indicates compensatory neuronal recruitment during early disease stages of low amyloid deposition (Sperling et al., 2010; Mormino et al., 2011; Ashraf et al., 2015), but the overexposure of the brain to glucose might in turn promote amyloid deposition (Cohen et al., 2009), resulting in later neurodegeneration and hypometabolism. In line with this, Tg2576 mice feature brain glucose hypermetabolism at seven, but not at 19 months of age (Luo et al., 2012), and our 3×Tg mice showed lower metabolism during aging, together with a >15% reduction in brain mass. Third, our data document that brain responses to acute INI were blunted in 3×Tg-HFD mice, especially at 8 months. Summarizing these metabolic findings, middle-age 3×Tg-HFD mice were characterized by impaired cerebral glucose metabolism (compared to HFD mice) and cerebral insulin resistance (compared to ND, HFD, and 3×Tg-ND groups). Circulating PAI-1, i.e., a marker of metabolic syndrome and potential causal factor of Alzheimer’s pathology in humans and rodents (Oh et al., 2014; Bi Oh et al., 2015), was markedly elevated in 3×Tg-HFD mice, and was the sole negative predictor of brain glucose metabolism.

Brain insulin treatment has been suggested to counteract cognitive deterioration in rodents and humans (Marks et al., 2009; Craft et al., 2012; Anderson et al., 2017). One or few insulin injections improved memory, learning, neurodegeneration and/or insulin signaling in different studies and in a variety of models, whereas protracted insulin regimens have been limitedly explored, and have generated unclear results. In Long-Evans rats, an intracerebroventricular injection of 4 mU of insulin increased memory function after 24 h (Park et al., 2000). In 12-months old SAMP8 mice, one-single or 14 daily injections of INI improved cognitive performance (Salameh et al., 2015). In a mouse HIV-associated neurodegenerative (HAND) model, nine daily injections of 2.4 IU enhanced the levels of glucose, ATP, ADP, phosphocreatine, and creatine in homogenized brain tissue (Nedelcovych et al., 2018). In F344 rats, insulin signaling was improved 2 h after a single INI injection (0.075 IU zinc-free insulin formulation), but no cognitive improvement was seen, even after prolonging treatment for 9-days (Anderson et al., 2017). In adult male C57BL6/J mice, short- and long-term memory, and odor discrimination were improved after acute (Marks et al., 2009) but not chronic INI treatment (twice daily for 30–60 days), and insulin signaling was blunted in the chronic arm (Bell and Fadool, 2017). Consistent with these negative outcomes, continuous (12-week) ICV insulin infusion impaired synaptic plasticity in Wistar rats (Kamal et al., 2012), and the prolonged exposure of neuronal cell cultures to insulin provoked neuronal insulin resistance (Nazarians-Armavil et al., 2013). We are aware of few reports addressing the effects of INI in 3×Tg mice or similar genetic models of AD pathology. Chen et al. (2014) showed that seven daily INI (1.75 IU) vs. vehicle injections restored brain insulin signaling, increased the levels of synaptic proteins, and reduced Aβ40 levels and microglia activation in an early disease stage, i.e., in 9 months 3×Tg mice. Still addressing early AD pathologies, Mao et al. (2016) found that 6 weeks of INI therapy (1.0 IU per day) improved cognitive deficits and insulin signaling, reduced Aβ production and amyloid plaque burden, and increased neurogenesis in young, i.e., 4.5-months old APPswe/PS1dE9 [amyloid precursor protein (APP)/PS1] mice. Finally, one report in 3×Tg-HFD mice showed that a single peripheral insulin injection reversed the deleterious effects of HFD on memory (intraperitoneal insulin injection) and soluble Aβ42 levels (intravenous insulin injection) (Vandal et al., 2014). In spite of a high degree of heterogeneity among studies, rodent models and intervention protocols, the above observations, together with the findings that chronic INI therapy is more effective at lower than higher dose regimens in humans with MCI or Alzheimer’s disease (Craft et al., 2012), support the possibility that an excessive and continuous exposure of the brain to insulin may lead to a downregulation of insulin action. The novelty of our study is threefold. First, we used weekly administrations of INI to reduce continuity in brain overexposure to insulin; second, we tested the efficacy of chronic INI on cognition and hippocampal dimensions in 3×Tg-HFD mice, and examined different age groups, reflective of early and late AD pathology; third, we addressed the *in vivo* response of brain metabolism and cerebral insulin resistance to chronic INI in 3×Tg-HFD mice. An important finding in this study was that the efficacy of our weekly administration of INI was striking in magnitude and progressive over time, resulting in 3×Tg-HFD mice with entirely normal cognitive function. INI also normalized functional parameters of cerebral GU and insulin sensitivity, reducing peripheral glycemia and body weight. INI had a strong impact on hippocampal morphology, completely preventing the degeneration of the dentate gyrus and thinning of the granule cell layer of the infrapyramidal blade seen in untreated 3×Tg-ND and 3×Tg-HFD mice. The histological pattern was similar, and strongly associated to the number of alternation triplets observed in Y-Maze testing. The above finding is consistent with the neurogenesis shown in the dentate gyrus of 2×Tg (APP/PS1) mice (Mao et al., 2016) after 6 weeks of INI vs. vehicle treatment. Of note, the degree of preservation in cognition mirrored the decline in PAI-1, which was progressively normalized in INI treated mice. Instead, INI did not improve the deficit in glucose FE by the brain or the whole brain mass, which remained the earliest (FE) and latest (mass) non-modifiable hallmarks of the 3×Tg genetic background.

Our data confirm that 3×Tg mice overeat compared to controls (Adebakin et al., 2012; Knight et al., 2012), independent of diet type. Leptin deficiency in this model may partly explain this behavior. In Tg2576 mice, leptin deficiency was ascribed to reduced adiposity (Ishii et al., 2014), consistent with our observation of low adipokines in 3×Tg mice. Interestingly, leptin plays an important role in the pathogenesis of human dementia, and has been tested as therapy (Greco et al., 2010). However, leptin was not affected by chronic INI therapy, despite reduced feeding and improved cognition. One possibility is that leptin sensitivity in hypothalamic circuits was restored by INI (despite persistence of low circulating levels), considering that amyloid deposition can disturb arcuate NPY neuronal responses (Ishii et al., 2014). Alternatively, the 3×Tg model is characterized by high peripheral metabolism and thermogenesis (Knight et al., 2012, 2013) and by an impairment in gut-to-brain satiety regulation (Adebakin et al., 2012); both of these factors can stimulate food intake, but their response to chronic INI was not explored in our study. Finally, cognitive preservation may exert a direct effect to limit food intake due to improved memory, as lesions to cognitive areas cause hyperphagia (Cao et al., 2007; Davidson et al., 2009).

Study limitations include the use of anesthesia that may underestimate metabolism, but cannot be avoided during imaging, and the lack of molecular measures, since the study was meant to capture the phenotype in order to plan molecular evaluations. In addition, we did not administer INI to all groups, as this was an explorative study in the group with most severe conditions. We showed a complete normalization of cognition and hippocampal histology and metabolism in that group, but we cannot establish whether INI is more effective in 3×Tg-HFD vs. 3×Tg-ND or non-Tg mice.

## Conclusion

In conclusion, this study confirms our hypotheses that the 3×Tg background and HFD exert additive (genes*lifestyle) detriment to the brain, and that cognitive dysfunction is accompanied by an increase in appetite in 3×Tg mice. PAI-1 levels and leptin deficiency were identified as potential peripheral contributors. Chronic INI preserved cognition, preventing hippocampus tissue loss and normalized PAI-1 levels, alleviating cerebral metabolic abnormalities, also reducing food intake, body weight, and glycemia in 3×Tg-HFD mice.

## Ethics Statement

As stated in the manuscript, at the time this study was carried out, the law requested notification, without explicit approval, of the project to the Italian Ministry of Health, which serves as the official Ethical Authority for animal studies in Italy.

## Author Contributions

ES: data collection, contribution to data analysis and to manuscript drafting. MG: statistical analyses and results presentation, and contribution to manuscript drafting. VDS: histology data collection and analysis. DP, MT, MQ: PET-CT imaging and processing. PS: contribution to study design. SB: responsible for animal handling and wellbeing. PI: study design and coordination, data analysis, manuscript writing, and project funding. All the authors contributed to the revision of the manuscript and approved its submission for publication.

## Conflict of Interest Statement

The authors declare that the research was conducted in the absence of any commercial or financial relationships that could be construed as a potential conflict of interest.

## Footnotes

1 https://imagej.nih.gov/ij

## Abbreviations

3 × Tg: Triple transgenic (mice)
C: control (mice)
CT: computed tomography
^18^FDG: ^18^F-2-fluoro-2-deoxyglucose
FE: fractional glucose extraction
GU: glucose uptake
HFD: high-fat diet
ID: injected ^18^FDG dose
INI: intranasal insulin therapy
ND: normal diet
PET: positron emission tomography.
