# Supplementary material for: Combined Effect of Fatty Diet and Cognitive Decline on Brain Metabolism, Food Intake, Body Weight, and Counteraction by Intranasal Insulin Therapy in 3×Tg Mice
Source: Front Cell Neurosci. 2019 May 3;13:188. doi: 10.3389/fncel.2019.00188 (PMC6509878; doi:10.3389/fncel.2019.00188)
Supplement: Supplementary file 1 [file Data_Sheet_1.pdf]

Combined effect of fatty diet and cognitive decline on brain metabolism, food intake, body weight, and counteraction by intranasal insulin therapy in 3xTg mice

Elena Sanguinetti[1,2*], Maria Angela Guzzardi[1*], Daniele Panetta[1], Maria Tripodi[1], Vincenzo De Sena[1], Mauro Quaglierini[1], Silvia Burchielli[3], Piero A. Salvadori[1], Patricia Iozzo[1]

*Equal first authorship contribution

[1]Institute of Clinical Physiology, National Research Council (CNR), Pisa, Italy

[2]Scuola Superiore di Studi Universitari Sant'Anna, Pisa, Italy

[3]Fondazione Toscana Gabriele Monasterio (FTGM), Pisa, Italy

Correspondence to: Patricia Iozzo, MD, PhD, Institute of Clinical Physiology, National Research Council (CNR), via Moruzzi 1, Pisa, 56124, Italy; phone: +39-050-3152789, Email: patricia.iozzo@ifc.cnr.it.

Supplementary Material (SM)

SM Table 1: two-way ANOVA for the effects of diet and genotype in group comparisons in brain regions from PET imaging

	Diet	Genotype	Diet*Genotype
**8 months**			
Fractional extraction			
Frontal cortex	0.759	**0.002**	0.894
Medial cortex	0.956	**<0.0005**	0.779
Dorsal striatum	0.693	**<0.0005**	0.908
Globus pallidus	0.551	**<0.0005**	0.843
Thalamus	0.292	**<0.0005**	0.741
Hypothalamus	0.330	**<0.0005**	0.935
Hippocampus	0.520	**<0.0005**	0.733
Temporal cortex	0.922	**<0.0005**	0.502
Amygdala	0.482	**<0.0005**	0.784
Sensory cortex	0.730	**<0.0005**	0.570
Cerebellum	0.108	**<0.0005**	0.609
Glucose uptake			
Frontal cortex	**<0.0005**	0.274	0.426
Medial cortex	**<0.0005**	0.209	0.510
Dorsal striatum	**<0.0005**	0.054	0.330
Globus pallidus	**<0.0005**	**0.027**	0.298
Thalamus	**<0.0005**	**0.047**	0.372
Hypothalamus	**<0.0005**	0.064	0.261
Hippocampus	**<0.0005**	0.140	0.482
Temporal cortex	**<0.0005**	**0.038**	0.115
Amygdala	**<0.0005**	0.114	0.463
Sensory cortex	**<0.0005**	**0.033**	0.391
Cerebellum	**<0.0005**	**0.051**	0.470
**14 months**			
Fractional extraction			
Frontal cortex	0.342	**<0.0005**	0.661
Medial cortex	0.197	**<0.0005**	0.486
Dorsal striatum	0.421	**<0.0005**	0.600
Globus pallidus	0.598	0.093	0.780
Thalamus	0.331	**0.001**	0.939
Hypothalamus	0.622	**0.001**	0.754
Hippocampus	0.311	**0.003**	0.919
Temporal cortex	0.355	**0.009**	0.919
Amygdala	0.363	**0.009**	0.797
Sensory cortex	0.325	**<0.0005**	0.828
Cerebellum	0.429	**0.002**	0.862
Glucose uptake			
Frontal cortex	0.646	0.067	0.917
Medial cortex	0.409	0.115	0.932
Dorsal striatum	0.624	0.081	0.916
Globus pallidus	0.444	**0.001**	0.902
Thalamus	0.461	0.139	0.811
Hypothalamus	0.628	0.132	0.656
Hippocampus	0.429	0.244	0.804

Temporal cortex	0.441	0.509	0.801
Amygdala	0.460	0.205	0.990
Sensory cortex	0.533	0.099	0.788
Cerebellum	0.480	0.198	0.763

SM Table 2: Mixed design ANOVA for the effects of acute intranasal insulin on brain regions from PET imaging

	**Acute insulin**	**Acute insulin * diet**	**Acute insulin * genotype**	**Acute insulin * diet * genotype**
**8 months**				
Fractional extraction				
Frontal cortex	0.064	0.142	0.616	0.854
Medial cortex	**0.025**	0.794	0.265	0.469
Dorsal striatum	0.090	0.802	0.499	0.563
Globus pallidus	0.977	0.820	0.415	0.736
Thalamus	0.566	0.587	0.759	0.652
Hypothalamus	0.539	0.816	0.468	0.563
Hippocampus	0.877	0.875	0.679	0.559
Temporal cortex	0.203	0.310	0.292	0.134
Amygdala	0.573	0.965	0.885	0.369
Sensory cortex	**0.004**	0.077	0.057	0.101
Cerebellum	**0.011**	0.858	0.255	0.969
Glucose uptake				
Frontal cortex	**<0.0005**	0.197	**0.009**	**0.053**
Medial cortex	**<0.0005**	0.816	0.106	0.274
Dorsal striatum	**<0.0005**	0.698	**0.003**	**0.046**
Globus pallidus	**<0.0005**	0.981	**0.001**	**0.037**
Thalamus	**<0.0005**	0.785	**0.001**	**0.024**
Hypothalamus	**<0.0005**	0.793	**<0.0005**	**0.003**
Hippocampus	**<0.0005**	0.630	**0.001**	**0.028**
Temporal cortex	**<0.0005**	0.288	**0.001**	**0.001**
Amygdala	**<0.0005**	0.650	**0.003**	**0.059**
Sensory cortex	**0.001**	0.097	**<0.0005**	**0.001**
Cerebellum	**<0.0005**	0.405	**<0.0005**	**0.016**
**14 months**				
Fractional extraction				
Frontal cortex	0.245	**0.027**	0.577	0.233
Medial cortex	0.059	0.220	0.318	0.310
Dorsal striatum	0.122	**0.052**	0.858	0.093
Globus pallidus	0.385	**0.057**	0.781	**0.062**
Thalamus	0.613	**0.024**	0.599	0.134
Hypothalamus	0.723	0.152	0.220	0.103
Hippocampus	0.964	0.161	0.632	0.343
Temporal cortex	0.101	**0.011**	0.077	0.203
Amygdala	0.664	**0.056**	0.793	0.404
Sensory cortex	0.107	0.353	0.628	0.311
Cerebellum	0.062	0.158	0.223	0.315
Glucose uptake				
Frontal cortex	**<0.0005**	0.656	0.133	0.149
Medial cortex	**0.010**	0.538	0.589	0.178
Dorsal striatum	**<0.0005**	0.646	0.162	0.135
Globus pallidus	**<0.0005**	0.760	0.203	0.189
Thalamus	**<0.0005**	0.966	0.145	0.120
Hypothalamus	**<0.0005**	0.695	0.123	0.140

Hippocampus	**<0.0005**	0.843	0.185	**0.072**
Temporal cortex	**<0.0005**	0.845	0.989	**0.094**
Amygdala	**0.004**	0.936	0.335	0.328
Sensory cortex	**0.009**	0.477	0.494	0.124
Cerebellum	**<0.0005**	0.991	0.171	**0.080**

SM Figure 1

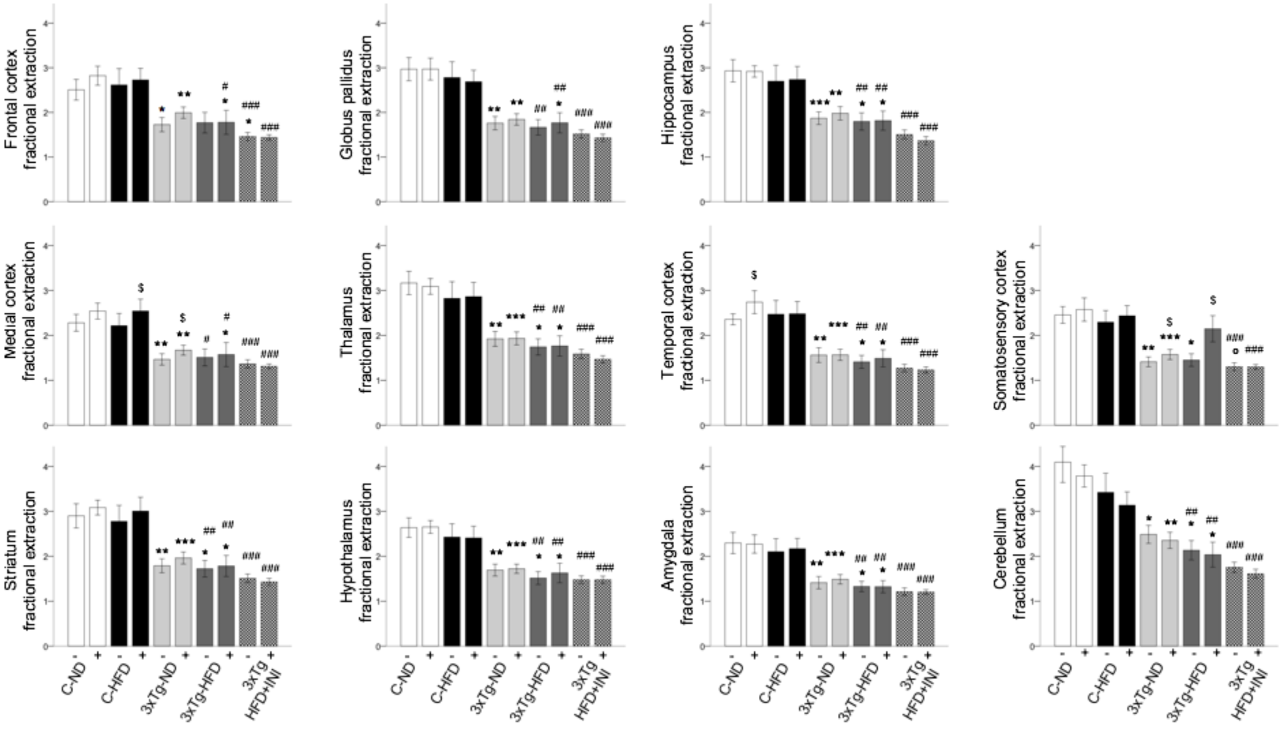

SM Figure 1. These data integrate results given in Fig. 6 of the main text, by primarily showing diet-induced increase in cerebral glucose uptake (GU) in all brain regions at 8 months of age, and the blunting effect of 3xTg background, together with the lack of response to acute intranasal insulin in 3xTg-HFD mice. Data are presented as mean±SEM. Sample sizes are given in Table 1. #p<0.05 vs ND control group (reference group), ^p<0.05 or less (^^) HFD vs ND (within-strain), ◦p<0.05 or less (°°) for treated 3xTg-HFD+INI vs untreated 3Tg HFD, $p<0.05 or less ($$) acute intranasal insulin vs baseline PET scans (-/+) (within-group, paired tests).

SM Figure 2

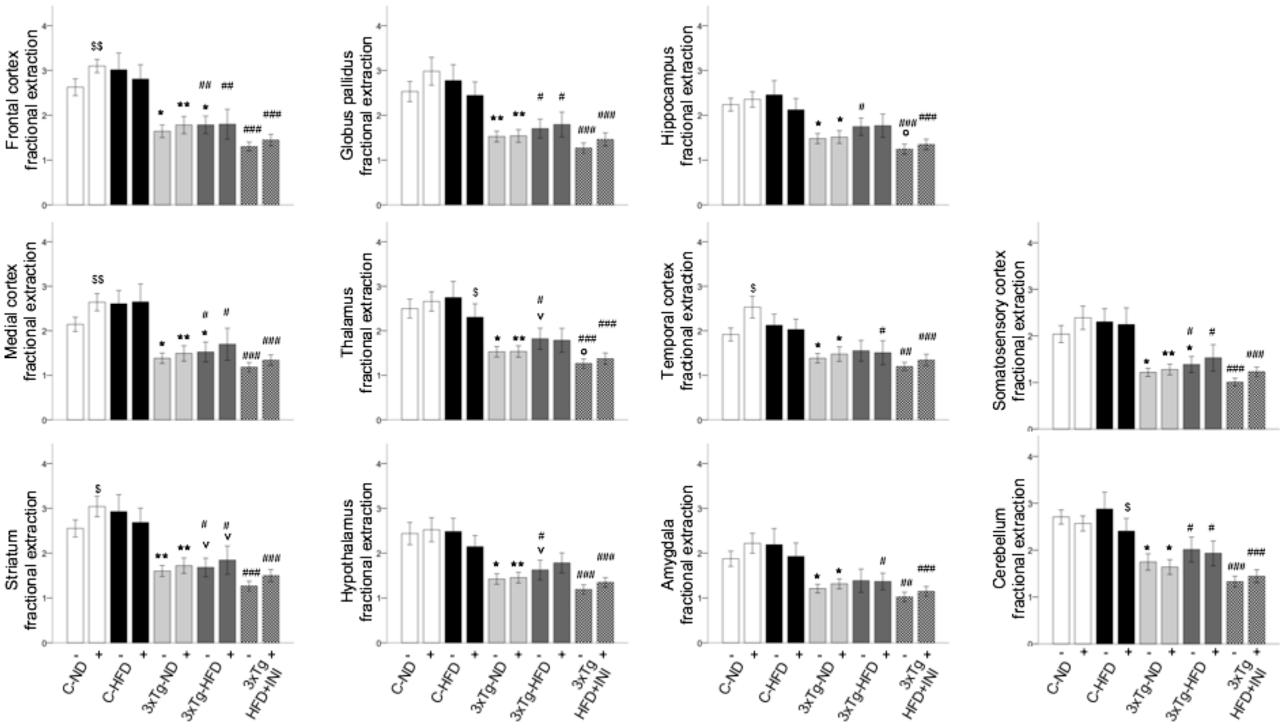

SM Figure 2. These data integrate results given in Fig. 6 of the main text, by showing a reduction in cerebral fractional glucose extraction involving all brain regions in all 3xTg groups at 14 months of age, and being mostly unaffected by HFD or chronic INI therapy. Acute intranasal insulin had very minor effects on fractional extraction. Data are presented as mean±SEM. Sample sizes are given in Table 1. *$p<0.05$ or less (**) or v$p=0.06$ ND-ND and HFD-HFD (between strains), #$p<0.05$ or less (##, ###) vs ND control group (reference group), ◦$p<0.05$ for treated 3xTg-HFD+INI vs untreated 3Tg HFD, $p<0.05 or less ($$) acute intranasal insulin vs baseline PET scans (-/+) (within-group, paired tests).

SM Figure 3

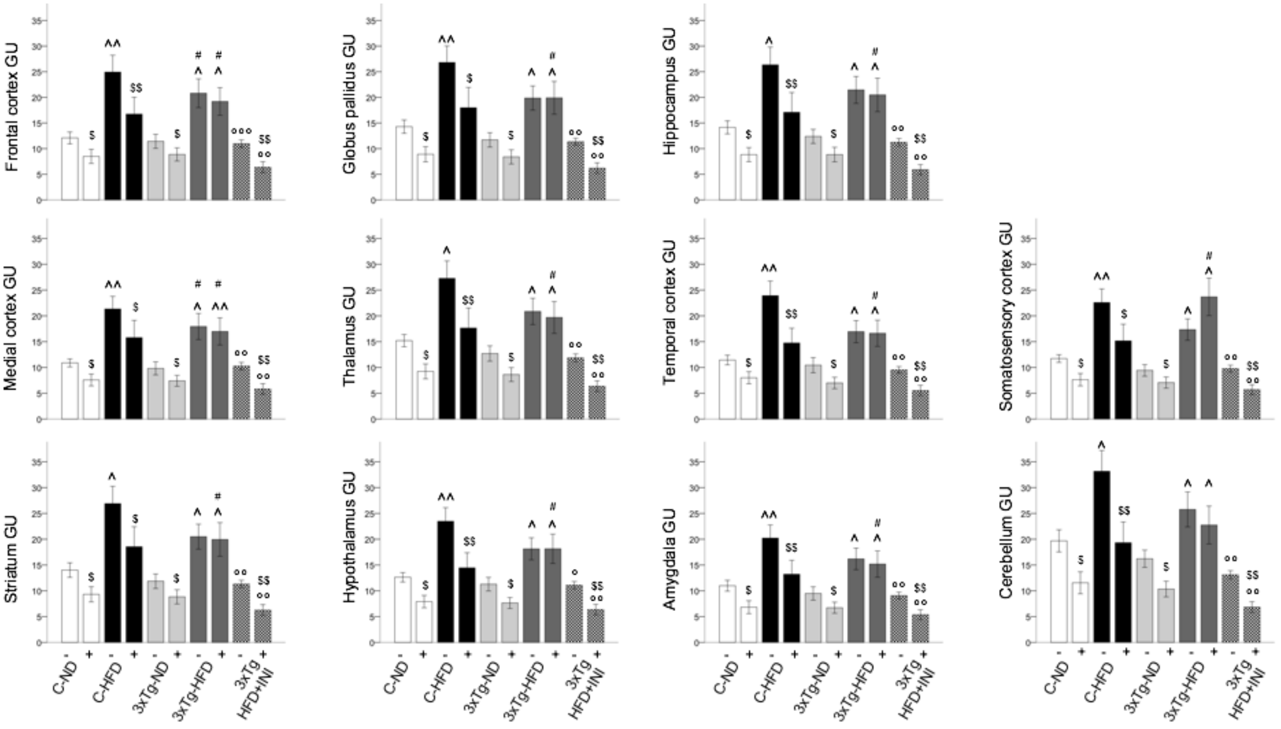

SM Figure 3. These data integrate results given in Fig. 6 of the main text, by primarily showing diet-induced increase in cerebral glucose uptake (GU) in all brain regions at 8 months of age, and the blunting effect of 3xTg background, together with the lack of response to acute intranasal insulin in 3xTg-HFD mice. Data are presented as mean±SEM. Sample sizes are given in Table 1. #p<0.05 vs ND control group (reference group), ^p<0.05 or less (^^) HFD vs ND (within-strain), ◦p<0.05 or less (°°) for treated 3xTg-HFD+INI vs untreated 3Tg HFD, $p<0.05 or less ($$) acute intranasal insulin vs baseline PET scans (-/+) (within-group, paired tests).

SM Figure 4

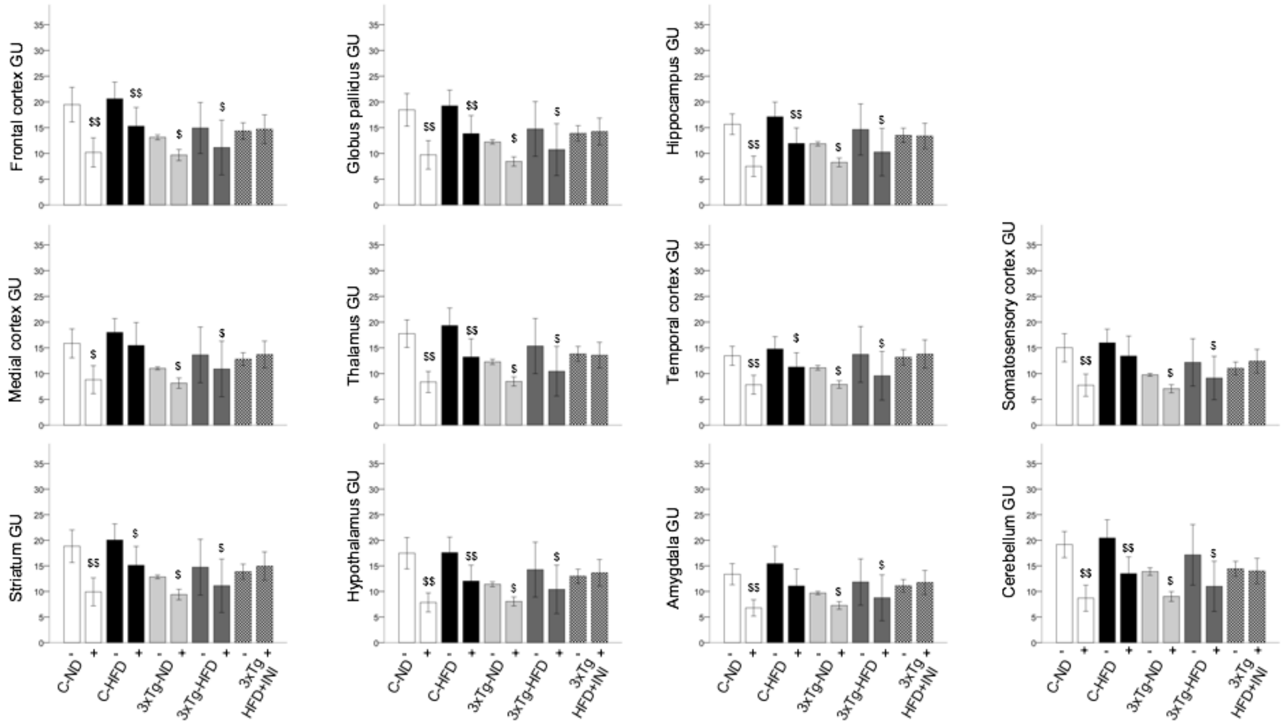

SM Figure 4. These data integrate results given in Fig. 6 of the main text, by primarily showing that a (non-significant) tendency towards reduced cerebral glucose uptake (GU) in 3xTg groups at 14 months of age, being mostly unaffected by HFD or chronic INI therapy. Acute intranasal insulin had more evident effects in ND mice, blunted effects in HFD and 3xTg-ND groups, and no effects in 3xTg-HFD+INI mice, indicating that cerebral insulin resistance, as induced by prolonged insulin treatment involves all brain regions. Data are presented as mean±SEM. Sample sizes are given in Table 1. \$p<0.05 or less (\$\$) acute intranasal insulin vs baseline PET scans (-/+) (within-group, paired tests).
